# Supplementary material for: Association of Nursery School-Level Promotion of Vegetable Eating with Caregiver-Reported Vegetable Consumption Behaviours among Preschool Children: A Multilevel Analysis of Japanese Children
Source: Nutrients. 2021 Jun 29;13(7):2236. doi: 10.3390/nu13072236 (PMC8308217; doi:10.3390/nu13072236)
Supplement: Supplementary file 1 [file nutrients-13-02236-s001.zip › nutrients-1220147-supplementary.pdf]

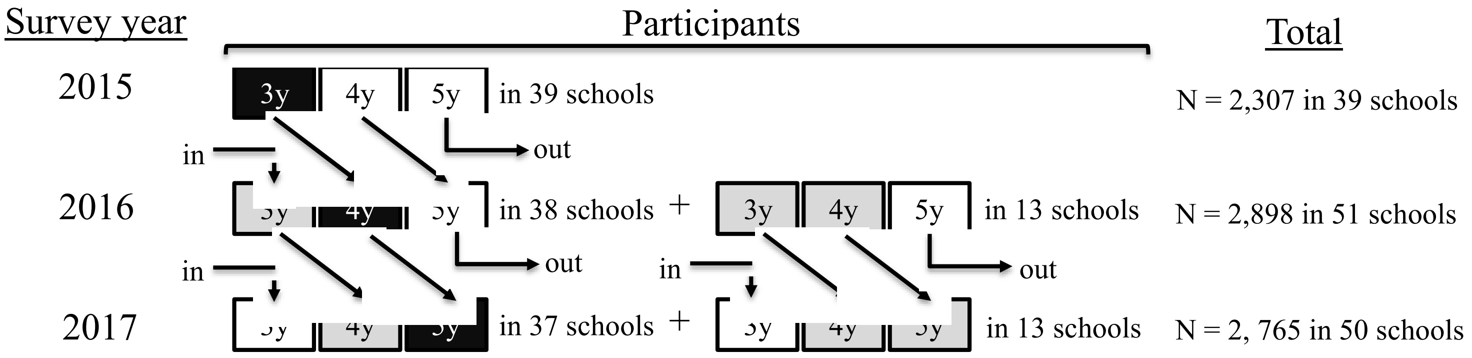

Scheme 1. Study design. Children in black boxes have been targeted three times. Children in gray boxes have been targeted two times.

Supplementary Table 1. Summary of the number of students and the school-level variable by school (*n* = 133)

	**2015**		2016		2017	
Nursery school number	***n***	**Ate vegetables first at meals (%)**	*n*	Ate vegetables first at meals (%)	*n*	Ate vegetables first at meals (%)
1	50	40.8	52	25.0	48	34.8
2	65	45.3	72	35.8	55	34.0
3	55	25.5	52	21.6	53	35.8
4	63	47.5	54	38.5	65	38.7
5	40	50.0	41	52.5	39	36.8
6	85	37.8	80	35.1		
7	57	27.3	45	36.4	50	32.0
8	51	37.3	47	55.3	51	46.0
9	59	39.7	73	44.4	71	50.0
10	48	42.6	40	35.9	47	26.1
11	64	31.7	62	33.3	65	33.3
12	75	41.1	74	48.6	75	44.0
13	52	50.0	48	43.8	54	38.9
14	63	33.9	61	43.1	59	31.6
15	30	48.3	30	53.8	28	17.9
16	58	36.8	47	28.9	58	41.1
17	68	39.4	67	38.1	65	36.9
18	72	28.6	75	33.8	82	47.6
19	57	54.5	77	45.2	87	50.0
20	57	28.6	56	51.9	57	35.1
21	78	46.7	76	44.7	80	46.3
22	51	50.0	54	40.8	52	45.1
23	61	29.3	72	25.0	58	24.1
24	50	26.0	59	41.4	56	48.1
25	46	41.3	42	33.3	41	43.9
26	83	36.3	81	31.6	69	35.3
27	55	27.8	51	31.9	44	37.2
28	54	37.7	56	46.3	37	43.2
29	54	38.5	56	46.3	54	59.3

30	45	45.5	57	46.3	53	37.7
31	57	45.5	68	37.1	68	39.4
32	46	39.1	42	56.1	53	44.2
33	67	43.3	70	30.4	65	31.3
34	77	23.3	97	38.9	96	48.9
35	84	23.2	93	26.4	94	28.9
36	57	19.6	61	9.8	71	23.9
37	36	33.3	54	28.8	46	23.9
38	67	39.4	52	28.8	56	37.0
39	67	31.3				
40			44	37.2	45	28.9
41			52	31.3	57	26.3
42			26	8.0	28	44.4
43			49	18.8	55	14.8
44			48	22.2	44	31.0
45			59	25.9	69	25.8
46			45	25.0	30	27.6
47			30	20.0	16	0.0
48			45	24.4	38	34.2
49			75	24.0	70	31.4
50			61	16.4	62	27.4
51			39	13.2	27	40.7
52			30	28.6	22	28.6
	Mean	Mean (SD)	Mean	Mean (SD)	Mean	Mean (SD)
Total	59	36.9 (8.6)	57	34.5 (10.8)	55	36.6 (9.6)

SD: standard deviation
